# Converting Equol Nonproducers to Producers: A Systematic Review of Randomized Clinical Trials

**DOI:** 10.1002/fsn3.72394

**Published:** 2026-09-27

**Authors:** Jiatong Li, Mengyi Li, Saied Aboushanab, Mark Ebeid, Rieko Hatanaka, Miho Iida, Masato Takase, Ian Jacobs, Arnur Gusmanov, Aya Hirata, Naoko Miyagawa, Naoki Nakaya, Daisuke Sugiyama, Curtis Tilves, Atsushi Hozawa, Xingnan Li, Noel T. Mueller, Panayiotis V. Benos, Akira Sekikawa

**Affiliations:** ^1^ Department of Epidemiology, School of Public Health University of Pittsburgh Pittsburgh Pennsylvania USA; ^2^ Ural Federal University Yekaterinburg Russia; ^3^ Computational and Systems Biology, School of Medicine University of Pittsburgh Pittsburgh Pennsylvania USA; ^4^ Tohoku Medical Megabank Organization Tohoku University Sendai Miyagi Japan; ^5^ Department of Preventive Medicine and Public Health Keio University School of Medicine Tokyo Japan; ^6^ Faculty of Nursing and Medical Care Keio University Fujisawa Kanagawa Japan; ^7^ Department of Epidemiology and Lifecourse Epidemiology of Adiposity and Diabetes (LEAD) Center University of Colorado Aurora Colorado USA; ^8^ University of Hawai’i Cancer Center Honolulu Hawaii USA; ^9^ Departments of Pediatrics and Epidemiology University of Colorado Aurora Colorado USA; ^10^ Department of Epidemiology, PHHP and COM University of Florida Gainesville Florida USA

**Keywords:** equol, producer, randomized clinical trials, soy, systematic review

## Abstract

Soy isoflavones have been associated with benefits for menopausal symptoms, cardiovascular health, and cognitive function, yet randomized clinical trials (RCTs) in Western countries have produced inconsistent results. A key factor may be equol, a metabolite of daidzein with stronger estrogenic and antioxidant effects, produced at lower rates in Western than in East Asian populations. This systematic review evaluates RCTs aimed at converting equol nonproducers to producers (CRD420251038428). We searched PubMed, Embase, and Web of Science for RCTs published between 1991 and 2025. Twelve RCTs (*n* = 730, 85.5% female) met inclusion criteria. Interventions included soy isoflavones alone, soy with probiotics, fermented soy products, seaweed, and wheat bran. Studies were conducted in Japan (*n* = 1), Brazil (*n* = 1), and Western countries (*n* = 10), with durations ranging from a single dose to 2.5 years. Study quality was assessed using the National Heart, Lung, and Blood Institute evaluation tool. None of the interventions significantly converted equol nonproducers into producers, with methodological limitations including small sample sizes, inconsistent equol measurement, and heterogeneous populations compromising reliability. Achieving equol producer status could unlock the full nutritional potential of soy isoflavones for menopausal, cardiovascular, and cognitive health. Future studies should prioritize standardized equol measurement, microbiome‐targeted interventions, and well‐powered, diverse populations to develop effective dietary strategies for Western populations where soy intake and equol production remain low.

## Graphical abstract

This systematic review, following PRISMA guidelines, screened 503 records identified through PubMed, Embase, Web of Science, and ClinicalTrials.gov, of which 12 randomized clinical trials (*n* = 730, 85.5% female) met inclusion criteria. Interventions included soy isoflavones alone, probiotics, or prebiotics combined with soy, fermented soy products, seaweed, and wheat bran, with durations ranging from a single dose to 2.5 years. None of the interventions significantly converted equol nonproducers into producers. Methodological limitations, including small sample sizes, inconsistent equol measurement methods, and heterogeneous study populations, compromised the reliability of findings. Future trials should prioritize standardized equol assessment with repeated measures, microbiome‐targeted interventions using equol‐producing bacterial strains, and adequately powered studies with diverse populations to develop effective strategies for enhancing equol production.

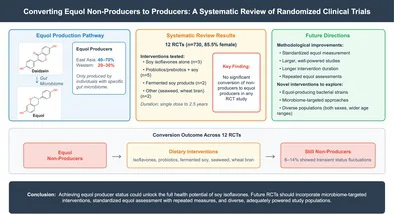

**Abbreviations**
FOS: fructo‐oligosaccharides
MeSH: Medical Subject Headings
NHLBI: National Heart, Lung and Blood Institute
NIH: National Institutes of Health
PRISMA: Preferred Reporting Items for Systematic Reviews and Meta‐Analyses
PROSPERO: International Prospective Register of Systematic Reviews
RCTs: randomized clinical trials
WISH: Women's Isoflavone Soy Health

## 1 Introduction

Nutritional interventions offer a promising and cost‐effective approach to addressing hormone‐related and cardiometabolic health challenges in the aging population (Giles et al. 2015). Soy isoflavones, a class of phytoestrogens with estrogen‐like activity, are considered promising natural compounds for alleviating menopausal symptoms and promoting cardiovascular and cognitive health (Igase et al. 2017; Bolaños et al. 2010). While observational studies suggest protective effects of soy isoflavones (Ma et al. 2020; Li et al. 2020; Ding et al. 2016), findings from randomized clinical trials (RCTs) have been inconsistent (Henderson et al. 2012). A key factor contributing to these inconsistencies may be the ability of the subjects to produce equol, a highly bioactive metabolite of the soy isoflavone daidzein, transformed by the gut microbiome in the intestine (Mayo et al. 2019). Equol, a selective estrogen receptor‐β agonist, has shown greater antioxidant activity and bioavailability than soy isoflavones. In both cell and in vivo studies, equol has been demonstrated to be permeable to the blood–brain barrier and to exert neuroprotective effects against neurotoxin‐induced cytotoxicity, supporting the potentiality of equol's neuroprotective function (Mayo et al. 2019). However, equol production is highly variable across populations, with up to 70% of East Asians producing it, partly due to a habitual soy‐rich diet, compared to only 20%–30% of Western populations (Kelly et al. 1995; Sekikawa et al. 2019). This variability may explain the inconsistent outcomes of soy isoflavone trials in European and US populations (Hodis et al. 2011).

Multiple observational studies in East Asians have linked equol but not soy isoflavones to favorable cardiovascular and neurovascular outcomes, such as lower aortic calcification (Zhang et al. 2022), and reduced risk of coronary heart disease and stroke. Equol producers have also been shown to have lower serum triglyceride levels, improved endothelial function, and reduced carotid intima‐media thickness compared to nonproducers (Sánchez‐Calvo et al. 2013). Furthermore, cross‐sectional studies have indicated that equol producers have a lower frequency of postmenopausal symptoms (Newton et al. 2015) and a lower risk of having mild cognitive impairment (Igase et al. 2017). A prospective cohort study in Chinese adults also showed that equol producers exhibited higher gut microbiota species richness and a distinct microbial composition compared to nonproducers, with enrichment of short‐chain fatty acid producing genera and depletion of inflammation‐associated taxa (Wu et al. 2022). These findings suggest that the health benefits associated with equol production may extend beyond the direct estrogenic activity of equol itself in the East Asian population, who have habitual soy intake, and may partly reflect a broader, functionally favorable gut microbial ecosystem. In vitro and animal model studies demonstrated that chronic exposure to soy isoflavone (Lu et al. 1996), prebiotics (Mustafa et al. 2020), probiotics (Elghali et al. 2012), and fermented soy products (Chien et al. 2006) increased equol bioavailability (de la Bastida et al. 2024), which makes these strategies worth investigating in broader populations to extend the cardiovascular and cognitive benefits of equol beyond habitual soy‐consuming populations.

Despite these promising findings, multiple pilot RCTs in humans aimed at converting equol nonproducers to producers have yielded mixed results in the past decades. This systematic review evaluates RCTs investigating interventions designed to promote equol production in nonproducers. We critically assess study methodologies, intervention types, outcomes, and study quality to identify key challenges and guide future research direction.

## 2 Methods

### 2.1 Literature Search

The review followed the Preferred Reporting Items for Systematic Reviews and Meta‐Analyses (PRISMA) guidelines (Page et al. 2021) and was registered at the International Prospective Register of Systematic Reviews (PROSPERO) website (CRD420251038428). We searched PubMed (1994 to January 2026), Embase (1994 to January 2026), and Web of Science (1998 to January 2026) for relevant studies. In PubMed, the following searching terms were used: (“Equol”[MeSH] OR “equol”[tiab]) AND (“Clinical Trial”[Publication Type] OR “Randomized Controlled Trial”[Publication Type] OR “clinical trial*”[tiab] OR “RCT”[tiab]). In Embase, we searched: (“equol”/exp. OR equol:ti,ab) AND (producer*:ti,ab OR nonproducer*:ti,ab OR “non producer*”:ti,ab OR convert*:ti,ab) AND (“clinical trial”/exp. OR “randomized controlled trial”/exp. OR random*:ti,ab OR intervention:ti,ab). In Web of Science, we searched: TS = (equol) AND TS = (producer* OR nonproducer* OR nonproducer* OR convert* OR phenotype* OR metabotype* OR “daidzein metabolism” OR “daidzein metabolite*”) AND TS = (trial OR randomized OR randomized OR intervention OR “clinical study”). Additionally, ClinicalTrials.gov was searched using “equol” limited to completed studies. Records from previous searches were identified. All records were imported into Zotero (https://www.zotero.org/) for screening.

### 2.2 Study Selection

The studies included in this systematic review met the following criteria: (1) RCTs; (2) included adult human participants (regardless of sex or menopausal status); (3) published as full text in English; (4) assessed equol or equol‐producing status before and after intervention. The following studies were excluded: (1) review papers and commentaries; (2) studies that could not obtain the full manuscript; (3) studies that did not have equol‐producing measurement data; (4) nonhuman clinical trials.

### 2.3 Outcome Definitions

The primary outcome was conversion of equol‐producing status, defined as a participant classified as nonproducer at baseline becoming classified as producer postintervention. Equol producer status was determined according to each study's criteria; where available, the log_10_‐transformed urinary equol/daidzein ratio > −1.75 was considered the reference standard (Setchell and Cole 2006). Secondary outcomes included changes in urinary or serum equol concentrations and the proportion of participants showing any status change during the study period.

### 2.4 Study Quality Assessment

All the studies from the database were screened by the researchers (J.L. and M.L.) according to the inclusion and exclusion criteria. After excluding duplicate and ineligible studies, the quality of all RCTs was independently assessed by two researchers (J.L. and M.L.) using the evaluation tool developed by the National Heart, Lung, and Blood Institute (NHLBI) (NHLBI, NIH, n.d.). This 14‐point evaluation questionnaire was answered with a “yes,” “no,” “not reported,” “cannot determine,” or “not applicable.” The RCTs were rated as “good,” “fair,” or “poor” by the researchers based on the criteria provided in this tool (Table S1).

## 3 Results

### 3.1 Searching Results

Of the 503 initial studies collected, 202 were excluded due to duplication. After screening the remaining 301 records, 192 were excluded for topic irrelevance, publication type (reviews/commentaries), or for not being human clinical trials. A full‐text review of the remaining 109 papers resulted in 91 exclusions due to a lack of equol‐producing status assessment or failure to evaluate this status both pre‐ and postintervention. An additional 6 observational studies were excluded as they were not RCTs (Table S3). Ultimately, 12 RCTs met all inclusion criteria and were included in the final assessments (Figure 1).

**FIGURE 1:**
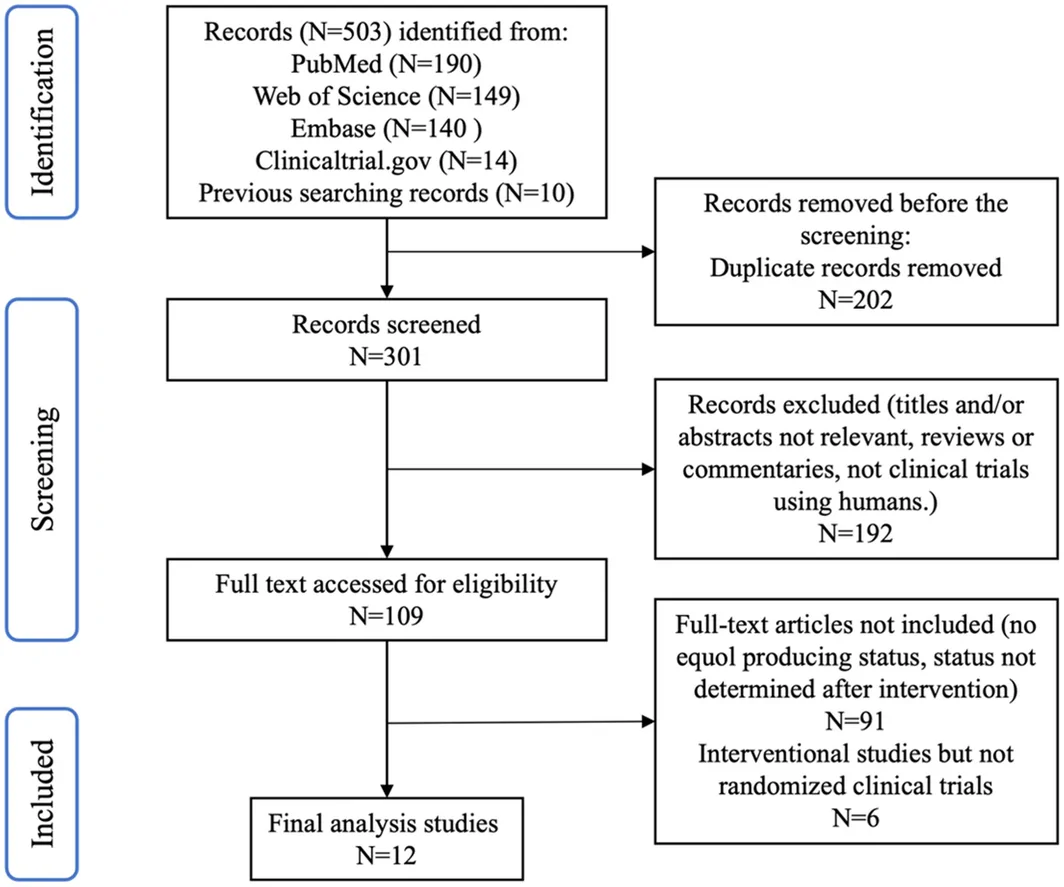
The flow chart for literature screening and reviewing.

### 3.2 Study Characteristics

The characteristics of the RCTs were extracted and summarized in Tables 1 and 2. A total of 730 participants (624 females) were enrolled at baseline, with 653 participants (597 females) completing the study and included in the final analysis (Table 1). Eight studies enrolled only female participants, including six that specifically targeted postmenopausal women. Only four studies performed the standardized soy challenge (Table S2), which involved having the participants consume soy isoflavone for three consecutive days before the baseline equol measurement (Setchell and Cole 2006). The interventions assessed across these trials included soy isoflavones alone (*n* = 3), soy combined with probiotics or prebiotics (*n* = 5), fermented soy products (*n* = 2), and other dietary components such as wheat bran or seaweed (*n* = 2) (Table 2). Study durations ranged from a single dose in 48 h to 2.5 years. Eight of the 12 trials employed a crossover design. In the quality assessment, seven studies were rated as “fair” quality and five studies as “poor” quality, with none meeting the criteria for “good” quality (Table 1).

**TABLE 1:** The characteristics and the evaluations of the RCTs.

Author and year	Country/Region	Sex (M/F)	Age (year)	Design	Dropout rate	Quality^a^
Lampe et al. (2001)	US	26F	20–40	CO	27%	Poor
Wiseman et al. (2004)	UK	38F/38M	18–45	P	0%	Fair
Nettleton et al. (2004)	US	40F	50–64	CO	8%	Fair
Bonorden et al. (2004)	US	37F	18–36	SB, P	8%	Fair
McMullen et al. (2006)	US	39M	18–37	SB, P	21%	Poor
Larkin et al. (2007)	Australia	15F/21M	49–72	CO	14%	Fair
Tsangalis et al. (2007)	Australia	16F	49–61	DB, CO	0%	Fair
Teas et al. (2009)	US	33F	50–70	DB, CO	55%	Poor
Franke, Lai, Pagano, et al. (2012)	US	96F	18–50	CO	15%	Fair
Franke, Lai, Halm, et al. (2012)	US	350F	60–68	DB	23%	Poor
Tousen et al. (2013)	Japan	43F	49–58	CO	21%	Poor
Silva et al. (2020)	Brazil	10F/8M	19–32	DB, CO	0%	Fair

Abbreviations: B, blinded; CO, crossover; DB, double‐blind; P, parallel; SB, single‐blinded.

^a^ Quality was assessed using the NHLBI evaluation tool. Studies rated “Fair” met more than 8 criteria, but lacked power calculation. Studies rated “Poor” had a dropout rate exceeding 20% or inadequate reporting of randomization.

**TABLE 2:** Intervention and the outcomes of the RCT studies.

Author and year	Interventions	Comparator	Duration	Soy challenge^d^	Equol assessment method	Outcome type^e^	Outcome
Soy isoflavone only							
Wiseman et al. (2004)	Isoflavone 104 ± 24 mg/day	0.54 ± 0.58 mg/day (crossover)	10 weeks	No	24 h urine	Conversion, Enhancement	No changes in equol producing status.
Franke, Lai, Halm, et al. (2012)	90 mg soy isoflavone/day	Placebo	2.5 years	No	Overnight urine	Conversion, stabilization	A high proportion of crossover equol‐producing status in the placebo group.
Franke, Lai, Pagano, et al. (2012)	50 mg isoflavone/day	Less than 10 mg isoflavone/day	6 months	Yes	Overnight urine/plasma	Conversion, stabilization	6%–11% of subjects changed equol‐producing status 1–2 times within the study period.
Probiotic and prebiotic combined with/without isoflavone							
Tousen et al. (2013)	37 mg isoflavone with 5 g fructo‐oligosaccharides/day	Sucrose (control) /day	2 weeks	No	Morning urine	Enhancement	No significant equol excretion changes.
Bonorden et al. (2004)	Probiotic capsule/day^b^	Placebo	57 ± 7 days	Yes	24 h urine	Conversion, enhancement	No significant equol excretion changes.
McMullen et al. (2006)	Probiotic capsules/day^b^	Placebo	57 days	Yes	24 h urine	Conversion, enhancement	No significant equol excretion changes.
Nettleton et al. 2004	44.4 ± 7.5 mg isoflavones with/without probiotics daily^a^	Milk protein with/without probiotics daily^a^	6 weeks	No	24 h urine and plasma	Enhancement	No urinary equol excretion changes.
Larkin et al. (2007)	Soymilk with/without probiotic daily^c^	Soy cereal with/without prebiotic daily	5 weeks	No	24 h urine/plasma	Conversion	None of the treatments induce equol formation in equol nonproducers.
Fermented soymilk and other supplements with isoflavones							
Lampe et al. (2001)	34 g soy protein with 16 g wheat bran daily	34 g soy protein daily	4 days/30 days	Yes	24 h urine	Enhancement	Do not significantly alter the capacity of producing equol.
Teas et al. (2009)	5 g of seaweed + soy isoflavone (2 mg/kg) daily	Soy isoflavone 2 mg/kg daily	7 weeks	No	24‐h urine	Enhancement	Equol secretion increased more with seaweed and soy isoflavone combination group.
Silva et al. (2020)	Soybean meal (SBM) daily	Fermented soybean meal (FSBM) biscuits daily	Single dose	No	48‐h urine	Conversion	No equol‐producing status change.
Tsangalis et al. (2007)	Fermented (FS) daily	Nonfermented soymilk (NFS) daily	14 days	No	urine	Conversion, enhancement	2 of the 8 subjects consuming FS changed to equol producers. No significant improvement in the equol‐producing status.

^a^ 10^9^ colony‐forming units *Lactobacillus acidophilus* DDS +1 and *Bifidobacterium longum* , 15–30 mg fructooligosaccharide.

^b^ 10^9^ colony‐forming units *Lactobacillus acidophilus* and *Bifidobacterium longum* strain DDS +1 and 10–15 mg fructooligosaccharide.

^c^ *Probiotic yogurts:* 10^8^ colony‐forming units/100‐g daily serving of each of Lactobacillus GG, *Lactobacillus acidophilus* , and *Bifidobacterium bifidus*; prebiotic bread: 16–20 g of resistant starch/serving.

^d^ Indicates whether a standardized 3 day soy isoflavone challenge was administered specifically to assess equol‐producer status, as distinct from the study intervention itself.

^e^ *Outcome type:* “Conversion” indicates assessment of whether equol nonproducers became producers; “Enhancement” indicates assessment of changes in equol excretion levels among existing producers; “Stabilization” indicates assessment of temporal consistency of equol‐producing status.

#### 3.2.1 Effects of Isoflavone‐Only Intervention and Equol‐Producing Status

In the studies that only used soy isoflavone as the intervention, Wiseman et al. measured the equol‐producing status at the intervention's midpoint and the study's endpoint in the high soy diet group (104 ± 24 mg isoflavones/day) (Wiseman et al. 2004). There were no statistically significant changes in equol‐producing status during the 5‐week high soy diet intervention. In the other two studies, which recruited only female participants with longer intervention (Franke, Lai, Halm, et al. 2012; Franke, Lai, Pagano, et al. 2012), the authors did not find that 6 months/2.5 years of soy isoflavone intake significantly changed the equol‐producing status. However, the data from multiple time point measurements showed that 14% of the postmenopausal and 11% of the premenopausal participants could have different equol‐producing status during the intervention period (Franke, Lai, Halm, et al. 2012; Franke, Lai, Pagano, et al. 2012). Moreover, in the secondary analysis of one of these studies—The Women's Isoflavone Soy Health (WISH) Study, which focused on postmenopausal women (Franke et al. 2010)—there was no correlation between antibiotic treatment and equol‐producing status in the 43 participants who took the antibiotic during the 2.5‐year study period (Franke, Lai, Halm, et al. 2012).

#### 3.2.2 Intervention With Probiotics and Prebiotics Combined With Isoflavone

Five RCTs used the probiotic components combined with soy isoflavones or probiotics only as the intervention. In a study of the postmenopausal Japanese population, 2 weeks of prebiotic intervention did not change the equol‐producing status or equol‐producing ability (Tousen et al. 2013). Bonorden et al. did a single‐blinded trial to determine if probiotic capsules could influence the equol‐producing status in healthy premenopausal women (Bonorden et al. 2004). However, in the two‐month intervention period, the equol‐producing status in the probiotic group did not change among equol nonproducers. Similar results were reported in the study that focused only on male participants (McMullen et al. 2006). In the study focusing on postmenopausal breast cancer survivors and healthy controls using the probiotic with soy supplement as a treatment, there were no changes in equol production before and after 6 weeks of treatment (Nettleton et al. 2004). However, 67% of the equol producers increased equol excretion when consuming probiotics, suggesting that probiotic intervention might enhance the equol‐producing ability in the equol‐producing women (Nettleton et al. 2004). Larkin et al. reported similar results, with 5 weeks of probiotic yogurt with soy did not change the equol‐producing status, but the equol‐producing ability was increased in the equol‐producer population, including male and postmenopausal females (Larkin et al. 2007).

#### 3.2.3 Intervention With Fermented Soymilk and Other Supplements With Isoflavones

The remaining four trials used wheat bran, seaweed with soy isoflavones, and fermented soymilk as interventions. Lampe et al. showed that a 1‐month wheat bran with soy protein treatment did not change the equol‐producing status or equol secretion levels in premenopausal women (Lampe et al. 2001). The double‐blind crossover trial that combined seaweed with soy by Teas et al. reported that 7 weeks seaweed could increase the equol excretion in the equol producers but did not change the equol‐producing status in the nonproducers (Teas et al. 2009). In the single‐dose trial using *S. cerevisiae* fermented soybean biscuits, Silva et al. found that compared to the nonfermented soybean biscuits, equol producers who took the fermented biscuits had a trend of excreting more equol (Silva et al. 2020). The results suggested that the fermentation might improve the isoflavone absorption and metabolism. However, in the trial focusing on the postmenopausal women who used bifidobacteria‐fermented soymilk as a treatment, there were no equol‐producing status changes or equol excretion changes in the research population after 14 days of treatment (Tsangalis et al. 2007).

## 4 Discussion

This systematic review of 12 RCTs demonstrates that current interventions have not been sufficient to convert equol nonproducers to producers. None of the studies reported statistically significant changes in equol‐producing status or the level of equol excretion. Although two trials reported a modest increase in equol production among existing equol producers with long‐term soy treatment (Tsangalis et al. 2007) and seaweed treatment (Teas et al. 2009), thus showing potential to enhance equol‐producing ability, these results did not reach statistical significance. Additionally, two studies found instability in equol‐producing status during the intervention in pre‐ and postmenopausal females (Franke, Lai, Halm, et al. 2012; Franke, Lai, Pagano, et al. 2012), suggesting that equol production may fluctuate rather than being a fixed trait, a finding that complicates study design and outcome assessment. To the best of our knowledge, this is the first systematic review to evaluate RCTs aimed at converting equol nonproducers to producers, identify key methodological challenges, and synthesize insights into diverse intervention strategies. This review provides a formal systematic quality assessment of these trials and consolidates the evidence base toward structured and actionable recommendations for future trial design.

The failure of most interventions to induce equol production likely reflects the ecological complexity of the gut microbiome, where equol conversion depends on the coordinated activity of a limited consortium of anaerobes such as *Slackia isoflavoniconvertens* (Matthies et al. 2012) and *Adlercreutzia equolifaciens* (Maruo et al. 2008). The absence or low abundance of these taxa, coupled with Western dietary patterns that are low in fermentable substrates, may constrain the metabolic network required for equol biosynthesis, even with soy or probiotic supplementation (Mayo et al. 2019). Beyond individual taxa, multiple studies have independently reported that equol producers exhibit higher gut microbiota diversity than nonproducers, with enrichment of short‐chain fatty acid producing genera such as Alistipes, Coprococcus, and Subdoligranulum, and depletion of inflammation‐associated taxa such as *Ruminococcus gnavus* group and Fusobacterium (Wu et al. 2022; Iino et al. 2026). Moreover, equol‐producing bacteria have been detected in both producers and nonproducers at comparable levels, and no significant increase in equol‐related gene copy numbers has been observed during isoflavone interventions (Mayo et al. 2019), indicating that the broader microbial community context, rather than the mere presence of specific equol‐converting taxa, may determine conversion capacity (Iino et al. 2026). These findings suggest that successful conversion may require interventions that reshape the gut microbiota toward a functionally supportive microbial community, rather than simply increasing daidzein substrate availability or administering individual probiotic strains. Importantly, the probiotic strains tested in the reviewed RCTs were predominantly *Lactobacillus acidophilus* and *Bifidobacterium longum* , which are not themselves equol‐converting organisms. While these strains possess β‐glucosidase activity capable of hydrolyzing isoflavone glucosides to aglycones (Sánchez‐Calvo et al. 2013), they cannot catalyze the subsequent reductive steps from daidzein to equol. Increasing substrate availability through aglycone release, therefore, does not ensure conversion to equol in the absence of specialized equol‐converting taxa.

The secondary analysis from the WISH trial suggested the instability of the equol‐producing status in female populations (Franke, Lai, Halm, et al. 2012; Franke, Lai, Pagano, et al. 2012). Consistent with this observation, a four‐year prospective cohort study of 498 Japanese adults reported that 26.1% of participants exhibited unstable equol‐producing status across annual assessments, despite comparable daidzein intake across groups (Iino et al. 2026), reinforcing the concern that single‐timepoint classification may misclassify participants in intervention trials. Importantly, stable equol producers in that cohort exhibited significantly higher gut microbiota diversity and a distinct microbial composition enriched in short‐chain fatty acid producing genera compared to both unstable producers and nonproducers (Iino et al. 2026), suggesting that only sustained equol production, supported by a functionally favorable gut microbial ecosystem, may confer the full health benefits attributed to equol. Unstable producers, whose intermediate microbiota profile suggests they may be more amenable to intervention than persistent nonproducers, represent a potentially important target population for future trials. Stratifying unstable producers as a distinct subgroup and incorporating stabilization of equol production as a trial endpoint alongside de novo conversion may increase the likelihood of detecting meaningful intervention effects.

Of the 12 RCTs reviewed, eight studies exclusively enrolled female participants, and six included only postmenopausal women, which raises concerns about the generalizability of the findings between males and females. Evidence from nonrandomized interventional studies further supports this concern. Lu et al. observed conversion in 3 out of 5 young female participants (Ages 15–39) following 33 days of soy isoflavone treatment (Lu and Anderson 1998), whereas a Japanese study reported conversion in only 2 out of 10 male participants after 3 months of treatment (Tanaka et al. 2009), suggesting that sex differences may affect equol production capacity. Additionally, equol production appears to be influenced by age, menopausal status, and gut microbiota composition (Santos‐Marcos et al. 2018; Badal et al. 2020), highlighting the need for more diverse study populations in future clinical trials. Sex‐dependent differences in isoflavone absorption and conjugation that affect equol concentrations independently of the microbial conversion step have also been demonstrated in gnotobiotic rat models (Matthies et al. 2012), further supporting the importance of enrolling both sexes and wider age groups in future trials.

The 12 RCTs also differed substantially in intervention characteristics, including the type and purity of isoflavone formulations, dosage, duration, route of delivery, and whether interventions were provided as supplements, whole soy foods, or fermented soy products. These pharmacological differences likely affect gastrointestinal absorption, hepatic metabolism, circulating half‐lives, and ultimately the amount of daidzein reaching the colonic microbiota for conversion (Sánchez‐Calvo et al. 2013; Setchell and Cole 2006). For example, fermented soy products contain higher proportions of bioavailable aglycone forms compared to unfermented soy, which predominantly contains glucoside conjugates requiring bacterial hydrolysis before absorption (Sánchez‐Calvo et al. 2013). Such heterogeneity across interventions makes it difficult to determine whether null results reflect a true lack of biological effect, insufficient bioavailability of the active substrate, or insufficient intervention duration. Future trials should carefully characterize and report the pharmacokinetic properties of the interventions used to enable meaningful comparisons across studies.

The absence of power calculations in all but one study, coupled with dropout rates exceeding 20% in five studies (Table 1), undermines the reliability of the findings. Studies rated as poor quality in this review received that rating primarily due to high dropout rates (Teas et al. 2009), which introduces potential attrition bias but does not invalidate the study findings. Excluding these studies would reduce an already small evidence base and could itself introduce selection bias; quality ratings are therefore reported transparently to allow readers to weigh the evidence accordingly. Inconsistencies in the methods used to determine equol‐producing status and define the cut‐off value further complicate the interpretation and comparability of results across studies. While most studies relied on 24‐h urine samples, others used overnight urine or serum samples, introducing variability in measurement approaches (Table S2). Only one trial used the log_10_‐transformed urinary equol/daidzein ratio with the cut‐off value as > −1.75, a method shown to reliably distinguish equol‐producers (Setchell and Cole 2006). Notably, the Franke et al. postmenopausal WISH trial (Franke, Lai, Halm, et al. 2012) observed status crossover even among participants in the placebo group, suggesting that equol‐producing status may fluctuate independently of specific intervention in some individuals. This instability implies that single‐timepoint classification of producer status, as used in most of the reviewed trials, may misclassify participants, and future trials should incorporate repeated equol assessments to improve classification accuracy (Franke, Lai, Halm, et al. 2012; Franke, Lai, Pagano, et al. 2012). Moreover, the standardized soy challenge test, where participants consumed soy products for three consecutive days to assess equol‐producing status, was employed in only four clinical trials (Lampe et al. 1998; Setchell et al. 2002), limiting the consistency and robustness of these assessments.

In the RCTs, soy isoflavone treatment lasting at most 10 weeks did not show a statistically significant effect on equol‐producing status. However, nonrandomized interventional studies suggest that longer‐term soy treatment may have greater potential to induce conversion (Table S3). In three of five non‐RCT studies using soy isoflavone as an intervention, soy isoflavone intake successfully converted equol nonproducers to producers (Lu and Anderson 1998; Tanaka et al. 2009; Ko et al. 2010). One study that gave 1000 mL soy milk/day and continued for 16 weeks showed that 8 of the 20 equol nonproducers successfully became equol producers (Ko et al. 2010), suggesting that long‐term and large amounts of soy intake might be an effective intervention. Future clinical trials may consider the longer intervention duration as well as higher dose to evaluate the effect of soy intervention.

As noted above, the β‐glucosidase activity of Lactobacillus and Bifidobacterium enables hydrolysis of isoflavone glucosides to aglycones and can increase daidzein content in fermented soymilk in vitro (Sánchez‐Calvo et al. 2013; Chien et al. 2006), but this step alone does not catalyze the subsequent reductive conversion to equol. Fructo‐oligosaccharides (FOS), the prebiotic which could stimulate the growth of bifidobacteria, showed the ability to improve equol‐producing status in the animal model (Ohta et al. 2002). In the in vitro study, isoflavone could be effectively transformed into equol by combining FOS and *Bifidobacterium longum* (Mustafa et al. 2020). However, neither probiotics nor prebiotics intervention significantly affected the conversion of equol nonproducers to producers from the RCTs (Tousen et al. 2013; Bonorden et al. 2004; McMullen et al. 2006; Larkin et al. 2007). Similarly, a pilot study in postmenopausal women found no statistically significant correlation between bacterial phyla and urinary equol excretion after 16 weeks of combined isoflavone and probiotic treatment following correction for multiple comparisons (Ribeiro et al. 2025). These results suggested that short‐term probiotics or prebiotics treatment alone might not be able to affect the equol‐producing status. Future trials should prioritize equol‐converting organisms such as *Slackia isoflavoniconvertens* (Matthies et al. 2008) or *Adlercreutzia equolifaciens* (Maruo et al. 2008), potentially in combination with prebiotic substrates that selectively support their colonization over extended intervention periods. *S. isoflavoniconvertens* has been demonstrated to stably colonize the intestine and convert daidzein to equol in vivo in a gnotobiotic rat model harboring a simplified human microbiota (Matthies et al. 2012), providing direct preclinical evidence supporting its prioritization in future human trials.

Equol‐producing status appears to depend not only on the presence of equol‐converting bacteria but also on their abundance and metabolic activity. Japanese population studies detected equol‐converting bacteria in both producers and nonproducers, yet the abundance of specific taxa differed significantly between groups, and long‐term daidzein intake was associated with producer status (Yoshikata et al. 2019; Iino et al. 2019). Complementary evidence from European studies revealed phenotypic differences in fecal microbiome metabolism, with equol‐producing samples generating less O‐desmethylangolensin (Curiel et al. 2025) and showing greater microbial population expansion during soy interventions (Lira et al. 2015). These findings collectively suggest that simply harboring equol‐converting species is insufficient and that their abundance and metabolic activity within the broader microbial community may determine conversion capacity. This may partly explain why short‐term probiotic supplementation in the reviewed RCTs failed to shift producer status. Vegetarians in Western countries have been reported to produce equol at rates approximately four times higher than nonvegetarians, approaching those observed in Asian populations (Mayo et al. 2019; Setchell and Cole 2006). Plant‐based diets rich in dietary fiber and complex carbohydrates promote a gut microbial environment enriched in fermentative bacteria that support the daidzein‐to‐equol metabolic pathway (Setchell and Cole 2006), suggesting that dietary interventions extending beyond short‐term supplementation may be necessary to meaningfully alter equol‐producing status. Ribeiro et al. further observed that hormone therapy, but not isoflavone alone or isoflavone combined with probiotics, was associated with significant increases in bacterial genera involved in isoflavone metabolism in postmenopausal women (Ribeiro et al. 2025), suggesting that the hormonal milieu may also influence the gut microbial environment conducive to equol production. Differences in habitual dietary patterns between East Asian and Western populations likely contribute to these microbial differences, as long‐term soy consumption may sustain the equol‐converting microbial niche (Ko et al. 2010). Although 10 of the 12 clinical trials were conducted in Western countries, none included sufficient pre‐ and posttreatment fecal microbiome analyses, representing a critical gap that limits our ability to determine whether interventions altered microbial community structure even when they failed to induce equol production.

While the majority of observational evidence linking equol production to health benefits has been generated in East Asian populations with habitual soy intake (Igase et al. 2017; Ahuja et al. 2017), findings in Western populations have been inconsistent and influenced by factors such as age, sex, and marital status. Among the limited Western studies examining equol‐related health outcomes, results have been mixed. Some trials in postmenopausal women found no significant differences in hyperlipidemia (Greany et al. 2004) or inflammatory biomarkers (Hall et al. 2005) between equol producers and nonproducers, while others observed trends toward improved endothelial function (Kreijkamp‐Kaspers et al. 2005) or significant triglyceride reduction only among equol producers (Acharjee et al. 2015). These inconsistencies may reflect not only differences in study design and population but also the low prevalence of equol producers in Western populations, which limits statistical power to detect producer‐specific effects. Although S‐equol supplements have been shown to alleviate menopausal symptoms in equol nonproducing postmenopausal women (Aso et al. 2012), direct supplementation requires continuous use, whereas promoting endogenous equol production could provide sustained equol exposure from habitual dietary soy intake and may also reflect a broader, functionally favorable gut microbial ecosystem (Wu et al. 2022; Iino et al. 2026). This underscores the clinical importance of developing effective conversion strategies, as increasing the proportion of equol producers in Western populations could substantially enhance the efficacy of soy‐based interventions. Currently, an ongoing clinical trial in the US (NCT05741060) is using equol to improve cardiovascular and brain health in aging populations (Sekikawa et al. 2025). The baseline measurement of equol‐producing status in the trial could provide valuable insights into the relationship between equol production capability and potential health benefits in the US population, which has been less extensively studied compared to Asian populations.

This review has several limitations. The restriction to RCTs means that nonrandomized and observational evidence is discussed for biological context and mechanistic interpretation only. The small number of eligible trials (*n* = 12) and their heterogeneity precluded quantitative meta‐analysis. The reviewed trials were predominantly conducted in Western populations and enrolled mostly postmenopausal women, limiting generalizability. The quality of the included studies was generally low to moderate, with most rated as poor or fair primarily due to high dropout rates and the absence of power calculations.

In conclusion, currently tested interventions in RCTs have not been sufficient to induce conversion from equol nonproducer to producer status, likely due to inadequate statistical power, heterogeneous methodologies, and the absence of a standardized equol assessment protocol. Moreover, the limited incorporation of gut microbiome analyses restricts our understanding of the biological mechanisms underlying equol conversion. These negative findings should not be interpreted as evidence that conversion is biologically impossible, given that longitudinal observational data indicate equol‐producing status can change over time (Iino et al. 2026) and nonrandomized studies have reported successful conversion with prolonged soy exposure (Lu and Anderson 1998; Tanaka et al. 2009; Ko et al. 2010). Future RCTs should enroll both sexes across a range of ages with extended intervention durations (Setchell et al. 2002), incorporate equol‐converting probiotic strains (Matthies et al. 2012) rather than general‐purpose probiotics, and include systematic pre‐ and postintervention fecal microbiome analyses. Future trials should employ shotgun metagenomics for taxonomic and functional profiling, targeted quantitative PCR for known equol‐converting bacteria, functional pathway prediction analyses, and metabolomic profiling of equol pathway intermediates. Future studies should also clearly distinguish among three related outcomes when assessing intervention effects, including conversion of nonproducers to producers, enhancement of equol production among existing producers, and stabilization of equol‐producing status in unstable producers. A standardized equol assessment protocol using the log_10_‐transformed urinary equol/daidzein ratio (Setchell and Cole 2006) with repeated measurements should be adopted to enable meaningful cross‐trial comparisons. Such studies are essential for optimizing soy‐based interventions, particularly in Western populations where equol production prevalence is low and the potential health benefits of conversion could be substantial.

## Author Contributions

**Jiatong Li:** conceptualization, writing – review and editing, writing – original draft, investigation, formal analysis, data curation, validation, visualization. **Mengyi Li:** methodology, investigation, writing – review and editing, data curation. **Saied Aboushanab:** writing – review and editing. **Mark Ebeid:** writing – review and editing. **Rieko Hatanaka:** writing – review and editing. **Miho Iida:** writing – review and editing. **Masato Takase:** writing – review and editing. **Ian Jacobs:** writing – review and editing. **Arnur Gusmanov:** writing – review and editing. **Aya Hirata:** writing – review and editing. **Naoko Miyagawa:** writing – review and editing. **Naoki Nakaya:** writing – review and editing. **Daisuke Sugiyama:** writing – review and editing. **Curtis Tilves:** writing – review and editing. **Atsushi Hozawa:** writing – review and editing. **Xingnan Li:** writing – review and editing. **Noel T. Mueller:** writing – review and editing. **Panayiotis V. Benos:** writing – review and editing. **Akira Sekikawa:** writing – review and editing, conceptualization, funding acquisition, supervision, project administration.

## Funding

This work was supported by National Institute on Aging (R01AG074971) and National Institutes of Health (R01HL169332).

## Conflicts of Interest

The authors declare no conflicts of interest.

## Supporting information


**Data S1:** Supporting Information.
**Table S1:** Quality assessment of the RCTs.
**Table S2:** soy challenging and equol measurement methods of the RCT studies.
**Table S3:** The characteristics and the results of the interventional studies.

## Data Availability

Data sharing is not applicable to this article as no datasets were generated or analyzed during the current systematic review.
