# Supplementary material for: Converting Equol Nonproducers to Producers: A Systematic Review of Randomized Clinical Trials
Source: Food Sci Nutr. 2026 Sep 27;14(10):e72394. doi: 10.1002/fsn3.72394 (PMC13617254; doi:10.1002/fsn3.72394)
Supplement: Supplementary file 1 [file FSN3-14-e72394-s001.docx]

**Supplementary tables**

**Stable1. Quality assessment of the RCTs**

| Assessment Criteria (No.)* | 1 | 2 | 3 | 4 | 5 | 6 | 7 | 8 | 9 | 10 | 11 | 12 | 13 | 14 | Quality |
| --- | --- | --- | --- | --- | --- | --- | --- | --- | --- | --- | --- | --- | --- | --- | --- |
| ^[1]^ Lampe et al., 2001 | Y | Y | Y | N | NR | NR | N | N | Y | Y | Y | N | Y | Y | Poor |
| ^[2]^ Wiseman et al., 2004 | Y | Y | Y | NR | NR | Y | Y | Y | Y | Y | Y | N | Y | Y | Fair |
| ^[3]^ Nettleton et al., 2004 | Y | Y | Y | CD | NR | Y | Y | Y | Y | Y | Y | N | Y | Y | Fair |
| ^[4]^ Bonorden et al., 2004 | Y | Y | Y | N | NR | Y | Y | Y | NR | Y | Y | N | NR | Y | Fair |
| ^[5]^ Mcmullen et al. 2006 | Y | Y | Y | N | NR | Y | N | Y | NR | Y | Y | N | Y | Y | Poor |
| ^[6]^ Larkin et al., 2007 | Y | Y | Y | N | Y | Y | Y | Y | Y | Y | Y | N | Y | Y | Fair |
| ^[7]^ Tsangalis et al., 2007 | Y | Y | Y | Y | Y | Y | Y | Y | Y | Y | Y | N | Y | Y | Fair |
| ^[8]^ Teas et al., 2009 | Y | Y | Y | Y | Y | Y | N | Y | Y | Y | Y | N | Y | Y | Poor |
| ^[9]^ Franke et al., 2012 | Y | Y | Y | N | Y | Y | Y | Y | Y | Y | Y | N | Y | Y | Fair |
| ^[10]^ Franke et al., 2012 | Y | Y | Y | Y | Y | Y | Y | Y | Y | Y | Y | N | Y | N | Poor |
| ^[11]^ Tousen et al., 2013 | Y | Y | Y | Y | Y | Y | N | Y | Y | Y | Y | N | Y | Y | Poor |
| ^[12]^ Silva et al., 2020 | Y | Y | Y | Y | Y | Y | Y | Y | Y | Y | Y | N | Y | Y | Fair |
| Y: yes; N: no; NR: not reported; CD: cannot determine.  *1. Was the study described as randomized, a randomized trial, a randomized clinical trial, or an RCT?  2. Was the method of randomization adequate?  3. Was the treatment allocation concealed?  4. Were study participants and providers blinded to treatment group assignment?  5. Were the people assessing the outcomes blinded to the participants' group assignments?  6. Were the groups similar at baseline on important characteristics that could affect outcomes?  7. Was the overall drop-out rate from the study at endpoint 20% or lower of the number allocated to treatment?  8. Was the differential drop-out rate (between treatment groups) at endpoint 15 percentage points or lower?  9. Was there high adherence to the intervention protocols for each treatment group?  10. Were other interventions avoided or similar in the groups?  11. Were outcomes assessed using valid and reliable measures, implemented consistently across all study participants?  12. Did the authors report that the sample size was sufficiently large to be able to detect a difference in the main outcome between groups with at least 80% power?  13. Were outcomes reported or subgroups analyzed prespecified?  14. Were all randomized participants analyzed in the group to which they were originally assigned? | | | | | | | | | | | | | | | |

**Stable2. soy challenging and equol measurement methods of the RCT studies**

| Publication | measurement sample | soy challenge method | Equol measurement methods | Equol producer definition |
| --- | --- | --- | --- | --- |
| Soy isoflavone only | | | | |
| ^[2]^ Wiseman et al., 2004 | 24h urine | 10 weeks 104±24 mg treatment | ^2^LC-MS | Producing equol >1000 nmol/24 h or >1μmol/24 h. |
| ^[9]^ Franke et al., 2012 | overnight urine | 2 x 250 mL/d soy milk on 3 consecutive days | ^2^LC-MS | Equol/diazine ratio >0.018, or absolute EQ value more than either 0.5 or 1.0 nmol/mg creatinine in urine |
| ^[10]^ Franke et al., 2012 | overnight urine/plasma | Daily 90mg isoflavone | ^2^LC-MS | EQ/DE ratio >0.018, or the concentration of equol is higher than either 0.5 or 1.0 nmol/mg in the urine, or either more than 5 nM or 10 nM in plasma |
| Probiotic and prebiotic combined with/without isoflavone | | | | |
| ^[11]^ Tousen et al., 2013 | morning urine | One day 22mg soy isoflavone | ^3^TR-FIA | Log (urinary equol/ daidzein) > −1.70. |
| ^[4]^ Bonorden et al., 2004 | 24h urine | 4-day 55±11 mg isoflavones/day | ^3^TR-FIA | 24h urine equol excretion greater than 900 nmol |
| ^[5]^ Mcmullen et al. 2006 | 24h urine | 4-day 63±9 mg isoflavones/day | ^4^GC-MS | 24h urine equol excretion greater than 1800nmol |
| ^[3]^ Nettleton et al., 2004 | 24h urine and plasma | Daily 44.4±7.5mg isoflavones +probiotic | ^3^TR-FIA | Plasma equol concentrations >15 nmol/L and urinary excretion >1500 nmol/24 h. |
| ^[6]^ Larkin et al., 2007 | 24h urine/plasma | daily 29 mg of daidzein + 55 mg of genistein + 4.5 mg of glycitein | ^1^HPLC | Equol-positive: consistently had equol in plasma or urine. |
| Fermented soymilk and other supplements with isoflavones | | | | |
| ^[1]^ Lampe et al., 2001 | 24h urine | 3 days 34 g soy protein treatment | ^1^ HPLC | Urine equol level >1mg/mL |
| ^[7]^ Tsangalis et al., 2007 | 24-h urine | daily 22.6, 37.4 and 67.3 μmol daidzein per 200 ml soy milk | ^1^HPLC | urinary excretion of equol was >1 μmol/day. |
| ^[8]^ Teas et al., 2009 | 48-h urine | 85 mg genistein, 50 mg daidzein, and 8 mg glycitein | ^2^LC-MS | The log_10_ of the equol:daidzein ratio value >-1.75 |
| ^[12]^ Silva et al., 2020 | urine | One day 0.44mg/kg weight isoflavone | ^1^HPLC-DAD-FLD system | not clear |
| ^1^ HPLC: High-performance liquid chromatography  ^2^LC-MS: Liquid chromatography-mass spectrometry  ^3^TR-FIA: time-resolved fluoroimmunoassay;  ^4^ GC-MS: Gas chromatography-mass spectrometry | | | | |

**sTable 3. The characteristics and the results of the interventional studies.**

| Publication | Country/ Region | Sex(M/F) | Age (Year) | Intervention | Duration | Outcome |
| --- | --- | --- | --- | --- | --- | --- |
| ^[13]^ Lu and Anderson, 1998 | US | 6F/6M | 15-39 | 80-210 mg isoflavone/day | 33 days | 3 of 5 equol non-producer females produced equol after the intervention. |
| ^[14]^ Védrine et al., 2006 | France | 12F | 52–73 | 100mg isoflavones/day | 30 days | No equol secretion changes in equol non-producer. |
| ^[15]^ Tanaka et al., 2009 | Japan | 28M | 30-59 | 60mg soy isoflavone daily | 3 months | 2 of 10 equol non-producers became equol producers after intervention. |
| ^[16]^ Ko et al., 2010 | Taiwan | 13F/7M | 22-61 | 1000mL soymilk weekly | 16 weeks | 8 of 20 nonproducers became equol producers. |
| ^[17]^ Lipovac et al., 2015 | Austria | 17M/F | 20-30 | 80mg of  red clover isoflavones with/ without ^*^FOS | Single dose/6 days | FOS showed no influence yet the trend of increased equol production. |
| ^[18]^ Guada-muro et al., 2015 | Spain | 16F | 48-61 | 80mg isoflavone concentrate per day | 6 months | No changes in equol non-producers. Microbial populations increased in the feces of the equol producers. |
| *FOS: Fructooligosaccharides | | | | | | |

**Reference**

[1]	J. W. Lampe, H. E. Skor, S. Li, K. Wähälä, W. N. Howald, C. Chen, *J Nutr* **2001**, *131*, 740–744.

[2]	H. Wiseman, K. Casey, E. A. Bowey, R. Duffy, M. Davies, I. R. Rowland, A. S. Lloyd, A. Murray, R. Thompson, D. B. Clarke, *The American Journal of Clinical Nutrition* **2004**, *80*, 692–699.

[3]	J. A. Nettleton, K. A. Greany, W. Thomas, K. E. Wangen, H. Adlercreutz, M. S. Kurzer, *J Nutr* **2004**, *134*, 1998–2003.

[4]	M. J. L. Bonorden, K. A. Greany, K. E. Wangen, W. R. Phipps, J. Feirtag, H. Adlercreutz, M. S. Kurzer, *Eur J Clin Nutr* **2004**, *58*, 1635–1642.

[5]	M. H. McMullen, J. M. Hamilton-Reeves, M. J. L. Bonorden, K. E. Wangen, W. R. Phipps, J. M. Feirtag, M. S. Kurzer, *J. Altern. Complement. Med.* **2006**, *12*, 887–894.

[6]	T. A. Larkin, W. E. Price, L. B. Astheimer, *Nutrition* **2007**, *23*, 709–718.

[7]	D. Tsangalis, G. Wilcox, N. P. Shah, A. E. J. McGill, L. Stojanovska, *Eur J Clin Nutr* **2007**, *61*, 438–441.

[8]	J. Teas, T. G. Hurley, J. R. Hebert, A. A. Franke, D. W. Sepkovic, M. S. Kurzer, *The Journal of Nutrition* **2009**, *139*, 939–944.

[9]	A. A. Franke, J. F. Lai, I. Pagano, Y. Morimoto, G. Maskarinec, *Brit J Nutr* **2012**, *107*, 1201–1206.

[10]	A. A. Franke, J. F. Lai, B. M. Halm, I. Pagano, N. Kono, W. J. Mack, H. N. Hodis, *The Journal of Nutritional Biochemistry* **2012**, *23*, 573–579.

[11]	Y. Tousen, M. Uehara, F. Abe, Y. Kimira, Y. Ishimi, *Nutr J* **2013**, *12*, 127.

[12]	F. Silva, T. C. Lemos, D. Sandôra, M. Monteiro, D. Perrone, *J Sci Food Agr* **2020**, *100*, 2991–2998.

[13]	L. J. Lu, K. E. Anderson, *Am J Clin Nutr* **1998**, *68*, 1500S-1504S.

[14]	N. Védrine, J. Mathey, C. Morand, M. Brandolini, M.-J. Davicco, L. Guy, C. Rémésy, V. Coxam, C. Manach, *Eur J Clin Nutr* **2006**, *60*, 1039–1045.

[15]	M. Tanaka, K. Fujimoto, Y. Chihara, K. Torimoto, T. Yoneda, N. Tanaka, A. Hirayama, N. Miyanaga, H. Akaza, Y. Hirao, *Prostate Cancer Prostatic Dis* **2009**, *12*, 247–252.

[16]	T.-F. Ko, H.-S. Tsai, S.-M. Lin, C.-D. Liu, S.-P. Learn, R. Y. Y. Chiou, *J Food Sci* **2010**, *75*, H306-310.

[17]	M. Lipovac, A. Pfitscher, S. Hobiger, T. Laschitz, M. Imhof, P. Chedraui, A. Jungbauer, *Fitoterapia* **2015**, *105*, 93–101.

[18]	L. Guadamuro, S. Delgado, B. Redruello, A. B. Flórez, A. Suárez, P. Martínez-Camblor, B. Mayo, *Front Microbiol* **2015**, *6*, 777.
